# Perceived quality of medical services at outpatient department of public hospitals in Dawro Zone, Southern Ethiopia

**DOI:** 10.1186/s12913-023-09178-0

**Published:** 2023-03-02

**Authors:** Legese Utino, Bezawit Birhanu, Nigusu Getachew, Berhane Megerssa Ereso

**Affiliations:** Department of Health Policy and Management, Faculty of Public Health, Health Institute, Jimma University, P.O. Box 378, Jimma, Ethiopia

**Keywords:** Perceived quality, Outpatient department, Client perception, Dawro zone public hospitals

## Abstract

**Background:**

Quality of care is fundamental to universal health coverage. Perceived quality of medical services is one of the most determining factors of modern health care service utilization. Between 5.7 and 8.4 million deaths are attributed to poor-quality care each year in low- and middle-income countries (LMICs), and up to 15% of overall deaths are due to poor quality. For instance, in sub-Saharan Africa (SSA), public health facilities lack basic facilities such as a physical environment. Hence, this study aims to assess the perceived quality of medical services and associated factors at outpatient departments of public hospitals in the Dawro zone, Southern Ethiopia.

**Methods:**

A facility-based cross-sectional study was conducted on the quality of care among outpatient department attendants of Dawro zone public hospitals from May 23 to June 28, 2021. A total of 420 study participants were included via a convenient sampling technique. An exit interview was used to collect data using a pretested and structured questionnaire. Then it was analyzed using the Statistical Package for Social Science (SPSS) version 25. Both bivariable and multivariable linear regressions were carried out. Significant predictors were reported at *p* < 0.05 with a 95% confidence interval.

**Result:**

with a 100% response rate. The overall perceived quality was 51.15%. Fifty-six percent of study participants rated perceived quality as poor, 9% as average, and 35% of participants rated it as good perceived quality. The highest mean perception result was related to the tangibility (3.17) domain. Waiting time less than one hour (β = 0.729, *p* < 0.001), availability of prescribed drugs (β = 0.185, *p *< 0.003), having information on diagnoses (illness) (β = 0.114, *p* < 0.047), and privacy maintained (β = 0.529, *p* < 0.001) were found to be predictors of perceived good quality of care.

**Conclusion:**

A majority of the study participants rated the perceived quality as poor. Waiting time, availability of prescribed drugs, information on diagnoses (illness), and provision of service with privacy were found to be predictors of client-perceived quality. Tangibility is the predominant and most important domain of client-perceived quality. The regional health bureau and zonal health department should understand the issue and work with hospitals to improve outpatient service quality by providing necessary medication, reducing wait times, and designing job training for health care providers.

## Background

Quality of care is the extent to which health services are provided to individuals to increase the likelihood of desired health outcomes and are consistent with current professional knowledge [1]. Donabedian defined medical service quality as the application of medical science and technology in a manner that maximizes its benefit to health without correspondingly increasing the risk [2, 3]. The quality of health care has been described by six factors, namely efficiency, effectiveness, efficacy, optimality, legitimacy, and equity. The disparity between a customer's expectations of a service provider's performance and their evaluation of the services received determines the quality of care [4, 5]. It can be differentiated into observed and perceived quality from the provider's or user's perspective, and it can also be assessed from different perspectives such as client perception, service provider, and facility manager, although client perception in quality of service is assumed to be more important than providing feedback about service areas of strength and weakness that need to be improved [6, 7]. According to the 2016 Ethiopian demographic health survey (EDHS) report, in the last 20 years, there has been a huge success in health infrastructure construction and health workforce development for the expansion of primary and secondary health care units in Ethiopia. However, the wide disparity in equity and quality of health care delivery across the region and within the region in terms of quality and equity has been observed. There are a lot of efforts made by the FMOH (Federal Ministry of Health) of Ethiopia to improve the quality of healthcare services and better health outcomes [8]. Between 5.7 and 8.4 million deaths are attributed to poor-quality care each year in low- and middle-income countries (LMICs), and up to 15% of overall deaths are due to poor quality [4]. For instance, in sub-Saharan Africa (SSA), public health facilities lack basic facilities such as a physical environment (water supply and hygiene). Most of the time, public health facilities are bypassed for lack of diagnostic facilities, drugs, and qualified health personnel. [9]. In the last two decades, different initiatives and efforts have been made to improve the quality of health services. However, healthcare services in public health facilities still lack some sort of quality-related concerns. [10]. Thus, it is vital to investigate providing quality client service in Ethiopia. For instance, OPD service quality in eastern Ethiopia seems to be low and the service delivery of federal referral hospitals is also not satisfactory, thus leading the service consumers to frustration. In addition, in Arsi Negele hospital, payment for the service and a long waiting time for laboratory results hampered the service quality given to consumers. [11–13]. Other studies from different parts of the country revealed that there are marked discrepancies in outpatient service quality and satisfaction. For instance, recent findings from different public hospitals like Jimma medical center, Adare General Hospital, and Yekatit 12 hospital were low in terms of quality and satisfaction [14–16]. Findings from Dawro zone Mareka district indicated that most of the OPD services provisions in the health facilities such as laboratory, triage, and pharmacy services are poor in terms of quality, thus hampering the satisfaction of the service users [17]. According to the Dawro zone health department 2019/2020 annual report, the OPD visit per person per year is 0.4, which is very low concerning utilization to attain a national target of 2020 outpatient visits per person [18, 19]. According to researchers’ understanding, little is known about the current perceived quality of medical service at OPD in the study area. Therefore, the purpose of this study is to assess the perceived quality of medical services at the OPD of Dawro zone public hospitals.

## Methods

### Study design, setting and period

A hospital-based cross-sectional study design was conducted in public hospitals in the Dawro zone from May 23 to June 28, 2021. The Dawro zone is one of 17 zones in the southern nation and nationality people’s region (SNNPR). The administrative center of Dawro zone is Tercha town, which is located 571 km away from Addis Ababa and 285 km away from the capital city of SNNPR (Hawassa). There are 10 districts and 2 administrative towns in the zone with functional 186 health posts, 21 health centers, and three public hospitals, namely Tarcha General Hospital, Gessa, and Tocha Primary Hospitals. There are a total of 1884 health professionals working in health facilities. Of those health professionals, 28 doctors and 116 nurses are in three public hospitals. In relation to health service utilization, especially outpatient visits in 2012 EFY, around 6815 clients got service in Tocha primary hospital, 9626 clients in Gessa primary hospital and 16,024 clients in Tarcha General hospital [20].

### Population

The source and study population were all adult clients who attended the out-patient departments of public hospitals in the Dawro zone and conveniently selected clients who came to the hospitals for adult general OPD services during the study period, respectively. All adult general OPD service clients whose age was greater than 18 years during the study period were included, and clients who were unable to respond due to disease conditions were excluded from the study.

### Sample size determination and sampling procedure

**Fig. 1:**
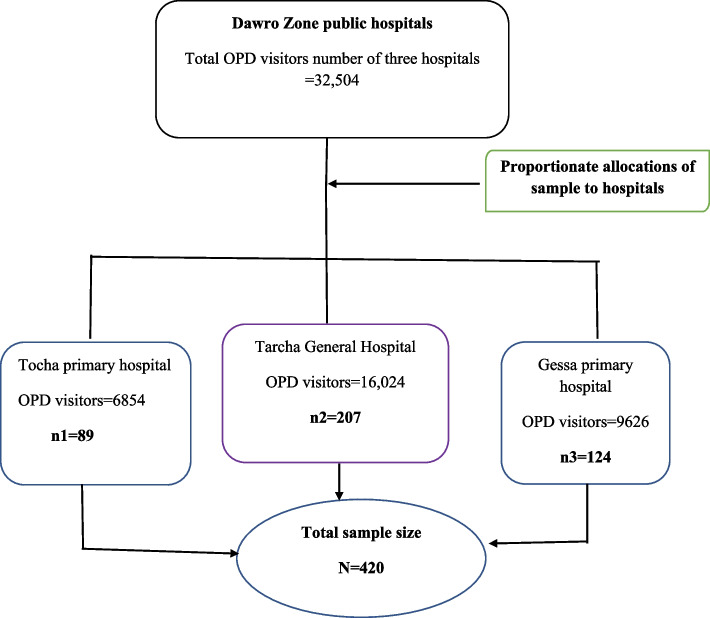
Schematic diagram of sampling procedure in perceived quality of medical services at outpatient department of public hospitals in Dawro zone

Quality of medical service at OPD to be 54.1% [11] other assumptions with 5% margin error (d) and confidence interval of 95% and by adding 10% non-response rate the final sample size becomes 420. The study participants were proportionally allocated to each hospital based on an OPD visitor number in 2019/2020 as per the Health management information system (HMIS) report. Twenty-one percent (89), 30% (124), and 49% (207) study participants were sampled from Tocha primary hospital, Gessa primary hospital, and Tercha general hospital, respectively (see Fig. 1).

### Study variables

The dependent variable was clients' perceived quality, and the independent variables were socio-demographic variables, health care provider-related variables, and health facility-related variables like waiting time, availability of prescribed and ordered medications, availability of investigations and laboratory service, availability of X-ray service, and opinion in service payment**.**

### Operational definitions

#### Client perceived quality

The quality of medical outpatient service in the perspective of clients measured by the SERVQUAL perception dimensions as follow. All 22 items in the scale to measure perceived quality together yield a maximum score of 110 and a minimum of 22.

$$\begin{array}{l}\mathrm{Percentagemeanscore}=\frac{\mathrm{Actualscore}-\mathrm{potencialminimum}}{\mathrm{Potentialmaximum}-\mathrm{potentialminimum}}\ast100=\left(\mathrm p1\%,\mathrm p2\%,\mathrm p3\%...............\mathrm p420\%/420\right)\left(29,30\right)\\\mathrm P\;-\;\mathrm{Represents}\;\mathrm{study}\;\mathrm{participants}.\end{array}$$

##### ***Poor perceived quality***

For those calculated percentage mean score result of the client's perceived quality of outpatient service is less than or equal to 50% (29).

##### ***Average perceived quality***

For those calculated percentage mean scores of the client perceived quality of outpatient service is between 51%—65% (29).

##### ***Good perceived quality***

For those calculated percentage mean scores of the dimension of client perceived quality of outpatient service is greater than or equal to 66% (29).

#### OPD

In this context**, **OPD means clients who are identified at central triage depending on their history and assigned to adult General OPD whose cases require medical intervention in a setting that does not involve an overnight hospital stay.

#### Medical service

Services including as the context requires confinement, treatments, procedures, tests, examinations, or other related services for the investigation or treatment of morbidity.

### Data collection instrument and procedure

The tools were adapted by reviewing different kinds of literature [7, 21–23]. The first part is socio-demographic which consists of age, sex, religion, ethnicity, educational status, marital status, occupation, place of residence, time from residency to hospital, mode of payment, average monthly income, and the number of visits. The second part is health facility-related questions which include waiting time, presence of ordered medications, presence of requested laboratory services, and presence of requested X-ray service, amount of payment, and opinion on payment. The third part is about healthcare provider-related questions consisting of courtesy and respect, communication, information on disease (illness), information on investigations and medication, satisfaction with the provider, opportunity to ask, received expected care, privacy, approach, and recommending the service to someone. The last part consists of the service quality dimension in the modified SERVQUAL tool namely tangibility, reliability, responsiveness, assurance, and empathy with their respective items. Data were collected by four diploma health professionals from adjacent health facilities and two BSc Nurse Supervisors using a structured questionnaire with a face-to-face exit interview.

### Data processing and analysis

Before being exported to SPSS version 25, the data were checked for completeness, edited, cleaned, coded, and entered into Epi-Data version 3.1. Descriptive statistics (including means, standard deviations, frequencies, and percentages) were calculated for socio-demographic and other variables and finally presented in texts and charts. A first percentage mean score was calculated based on the percentage of the maximum scale score. By using this continuous scale, bi-variable linear regression was conducted to determine the independent predictors of client perceived quality and to identify candidate variables for multiple linear regressions; a significance level of *p*-value < 0.25 was taken as a cut-off point for identifying candidates under bi-variable linear regression analysis. Multivariate linear regression analysis was conducted to identify independent factors associated with client perceived quality; a significant level of *p*-values less than 0.05 at 95% CI was taken as a cutoff point and an unstandardized Beta was used for interpretation. The final model was constructed using the backward elimination method. The items with a scale reliability coefficient (Cronbach’s alpha) of greater than 0.70 were considered.

## Results

### Socio-demographic characteristics

A total of 420 clients were enrolled in the study with a response rate of 100%. Out of this, 236 (56.2%) of the participants were males. The mean (± SD) age for the study participants was 42.94 (± 13) years, with a range of 18 and 75 years. A majority of 164 (39%) were those greater than 46 years. Regarding the Educational background of the participants, one-third (35.7%) completed primary education. As to the residents, 254(60.5%) were from rural areas. Most of the participants 202 (48.1%) were Protestants by religion. More than three fourth, (78.8%) of the participants were married. Almost one-fourth (24.5%) of the participants were farmers by occupation See Table 1.

**Table 1:** Socio demographic characteristics of participants on Perceived quality of medical service at OPD of public hospitals in Dawro Zone, 2021 *N* = 420

Variables	Category	Frequency	%
Age in years	< 25	53	12.7
	25—35	80	19
	36—45	123	29.3
	> 46	164	39
Sex	Male	236	56.2
	Female	184	43.8
Educational status	Unable to read	62	14.8
	Primary education	150	35.7
	Secondary education	114	27.1
	Graduated from college	94	22.4
Ethnicity	Dawro	351	83.6
	Wolayita	38	9
	Konta	12	2.9
	Kambata	9	2.1
	Amhara	10	2.4
Religion	Orthodox	191	45.5
	Protestant	202	48.1
	Catholic	24	5.7
	Muslim	3	0.7
Occupation	House wife	70	16.7
	Farmer	103	24.5
	Government employee	83	19.2
	Private business	96	22.9
	Student	13	3.1
	Merchant	55	13.1
Residence	Rural	254	60.5
	Urban	166	39.5
Marital status	Single	84	20
	Married	331	78.8
	Divorced	2	0.5
	Widowed	3	0.7
Mode of payment	In cash	280	66.7
	Insurance	134	31.9
	Organization	3	0.7
	Free	3	0.7
Number of visits in past 6 months	Once	138	32.9
	Twice	203	48.3
	Greater than or equal to three	79	18.8
Monthly Income	Less than or equal to 3000	252	60
	Greater than 3000	168	40
Time taken to get the hospital service in transport	Less than 30 min	217	51.7
	Greater than or equal to 30 min	203	48.3

### Health facility related factors

A majority (264, 62.9%) of the study participants waited more than one hour after having a card to be seen by the healthcare providers. Concerning the availability of drugs, only 129(29.3%) of study participants had got all prescribed drugs from the hospital’s pharmacy unit. Most (290, 66.7%) of the study participants have received their laboratory service in hospitals. X-ray service was ordered for 57(13.3%) of the study participants. Of those 48, (84.2%) of them had gotten the service at the facility. As to the opinion of the study participants concerning the payment for the service provided, 332(79%) of the study participants stated that the payment for the service was fair See Table 2.

**Table 2:** Health facility related variables of study on Perceived quality of medical service at OPD of public hospitals in Dawro Zone, 2021 *N* = 420

Variables	Frequency	%
Waiting time		
Greater than one hour	264	62.9
Less than one hour	156	37.1
Drugs prescribed and got		
All	129	29.3
Some	278	66.2
None	19	4.5
Laboratory test ordered		
Yes	377	89.9
No	43	10.1
Got ordered laboratory test in the hospital		
Yes	290	77
No	87	23
X- ray service Ordered		
Yes	57	13
No	363	87
Got x-ray service at the hospital		
Yes	46	84.2
No	11	15.8
Service payment		
Less than 225 Ethiopian Birr	195	46.4
Greater than or equal to 225 Ethiopian Birr	225	53.6
Opinion on payments		
Unaffordable	46	11
Fair	332	79
Cheap	42	10

### Health care provider related factors

More than half (281, 66.9%) of the participants responded that they have got respect and courtesy. Concerning communication, 280(66.7%) of the study participants said that they had effective communication with service providers. Nearly half (45.7%) of study participants reported that healthcare providers informed them of their diagnosis. Moreover, about half (213, 50.7%) of the study participants stated that the healthcare providers explained investigations and procedures to them See Table 3.

**Table 3:** Participants’ response on health care provider related factor on study perceived quality of medical service at OPD of public hospitals in Dawro zone 2021, *N* = 420

Variables	Frequency	%
The health care provider offered courtesy and respect		
Yes	281	66.9
No	139	33.1
The health care provider communicated effectively		
Yes	280	66.7
No	140	33.3
The health care provider explained the diagnosis (Illness)		
Yes	192	45.7
No	228	54.3
The health care provider explained Investigations and medications		
Yes	213	50.7
No	207	49.3
The health care provider provided an opportunity to ask him		
Yes	236	56.2
No	184	43.8
Satisfied with the behavior and accountability of health service providers in the OPD		
Yes	230	54.8
No	190	46.2
Privacy was maintained		
Yes	226	53.8
No	194	46.2
Received needed care at the right time		
Yes	232	55.2
No	188	44.8
Staffs have good approach		
Yes	233	55.5
No	187	44.5
Recommend the outpatient services to someone else		
Yes	254	60.5
No	166	39.5

### Perception of participants on quality of service

The perception dimensions of the clients were composed of five components, with a total of 22 items, and each item was measured on a five-point Likert scale. In our study, SERVQUAL perception has five dimensions, namely tangibility, reliability, responsiveness, assurance, and empathy. The mean scores of the five dimensions of perception of each domain were tangibility (3.17), empathy (3.15), assurance (3.07), responsiveness (2.905), and reliability (2.9). The mean score of perception dimensions in this study varied from 3.17 to 2.9, and the highest perception score was related to tangibility, while the lowest score was related to reliability. To determine the magnitude of client perceived quality, internal consistency (Cronbach’s Alpha) was first calculated for the items in the scale for measuring perceived quality. Accordingly, the items had a Cronbach’s alpha value of 0.964. All 22 items on the scale to measure perceived quality together yield a maximum score of 110 and a minimum of 22. A percentage mean score was

$$\mathrm{Percentage mean score}=\frac{\mathrm{Actual score}-\mathrm{potencial minimum}}{\mathrm{Potential maximum} - \mathrm{potential minimum}}*100= \left(\mathrm{p}1\mathrm{\%},\mathrm{ p}2\mathrm{\%},\mathrm{ p}3\mathrm{\%}...............\mathrm{p}420\mathrm{\%}/420\right)$$

where p- represents study participants.

**Fig. 2:**
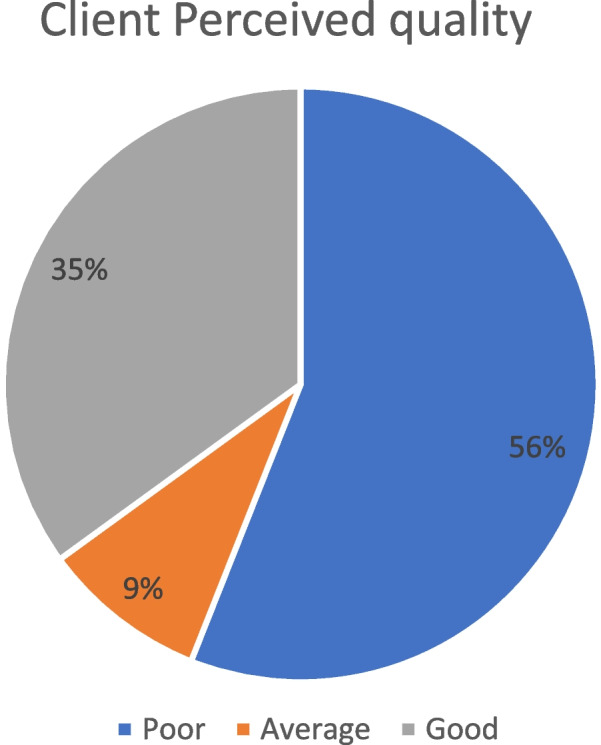
Client perceived quality of medical service at OPD of public hospitals in Dawro zone, 2021

Accordingly, client perceived quality (percentage mean score) with OPD services at Dawro zone public hospitals was 51.15%. Regarding classification based on their percentage mean score most 235 (56%) of the study participant’s rate as poor (see Fig. 2).

### Factors associated with client perceived quality

A total of fifteen (15) variables were candidates for multi-variable linear regression using the backward regression method. Of those four variables, two were statistically significantly associated with client perceived quality (p 0.05). Accordingly, waiting time, availability of drugs, information on diagnoses (illness), and privacy maintained were statistically associated with client-perceived quality. The variables in this model explained 77.4% (*R* = 0.880, R Square = 0.774, adjusted R Square = 0.771) of the variability in the client's perceived quality.

Accordingly, study participants with a waiting time of less than one hour to be seen by a healthcare provider have an average increment of client perceived quality of 0.729 (95% CI = 0.605, 0.853 *p* < 0.001) as compared to a waiting time greater than one hour. In terms of drug availability, participants who received all prescribed drugs from the hospital pharmacy unit had an average 0.185 (95% CI = 0.069, 0.322 p 0.003) increase in client perceived quality over those who received some. Also, our study showed that participants who had their diagnosis informed had a 0.114 (95% CI: 0.002 to 0.227 p0.047) unit increment in client perceived quality as compared to those who did not know.

The other predictor variable in our study is related to privacy. Participants whose privacy was maintained had an average 0.529 (95%CI: 0.412, 0.647, *p* < 0.001) increase in client perceived quality as compared to their counterparts See Table 4.

**Table 4:** Predictors of Client perceived quality of medical services at OPD of public hospitals in Dawro zone 2021, *N* = 420

S.no	Variables	Characteristics	Frequency	Unstandardized β Coefficients	*P*-value	95% Confidence Interval for β
**1**	Waiting time	> 1 h	236 (56.2)	1		
		< 1 h	184 (43.8)	0.729	** < 0.001**	(0.605, 0.853)
**2**	Drug availability	All	129 (29.3)	0.185	**0.003**	(0.069, 0.322)
		Some	278 (66.2)	1		
		None	19 (4.5)			
**3**	Provider explained diagnose	Yes	192 (45.7)	0.114	**0.047**	(0.002, 0.227)
		No	228 (54.3)	1		
**4**	Privacy was maintained	Yes	226 (53.8)	0.529	** < 0.001**	(0.412, 0.647)
		No	194 (46.2)	1		

Key 1—Reference’s category (compenents with highest frequency taken as reference categories)

## Discussion

The overall perceived quality of the present study is 51.15%. In this study, 35% of respondents rated the client's perceived quality as good. The finding was less than the studies done in Mexico, Nigeria (85.2%), and Tehran (57.5%) respectively [19, 24, 25]. Also, the finding was less than a study done in India Faridabad city which is (93.9%) [26]. The reason for this disparity could be a difference in setting in terms of economy, level of facilities, and quality of service provided [10]. Another reason could be the differences in way of measurement. For instance, the measurement for classification of client perceived quality in our study is based on the percentage mean score of total participants whereas other studies like Teheran and Nigeria used different categorical systems (i.e., Respondents were asked to rate their experience as good, average and poor) this could make a slight difference in the result. The other possible reason might be the difference in the study period.

The present study showed that the highest mean perception score was related to the tangibility dimension. The finding was similar to the study done at Woliata sodo referral hospital [27]. However, the finding was different from a study done in a federal police referral hospital, which found the highest mean perception score was related to the responsiveness dimension [19]. The finding was different from the study done in Bahrain, where the highest mean score in that study was related to the reliability dimension [28]. In addition, also Nepal’s study finding was different from our finding and the highest mean score was related to the assurance dimension [29].

The reason for this difference might be the number of human resources in the hospitals, and the differences between facility setting and professional composure. Another reason for the differences might be healthcare workers' attention and attitude toward their clients. In addition, the possible reason for this might be due to the health policy of the countries concerning client-centered service provision.

In our study, a long waiting time to be seen by a healthcare worker was one of the factors associated with a client’s perceived quality. Accordingly, as compared to study participants, those with a waiting time of less than one hour to be seen by a healthcare worker have an average increment of 0.729 (95% CI = 0.605, 0.853 *p* < 0.001) in client perceived quality than those who wait longer than one hour. Other studies also support this finding. According to a study conducted in Lagos, Nigeria, patients with short waiting time histories were 5.08 times more likely to be satisfied with the quality of care than those with long waiting times. [27]. This implies that long waiting time was associated with poor perceived quality. This long waiting time could be attributed to poor quality of care and a decrease in the number of clients.

Client-perceived quality was also an independent predictor of client perception. Accordingly, participants whose privacy was maintained had an average increase of 0.529 (95%CI: 0.412, 0.647 *p* < 0.001) in their client-perceived quality as compared to their counterparts. A study conducted in Iran found that clients whose privacy was not maintained rated service quality as lower than their counterparts [30]. This finding is supported by research from Nigeria's Ekiti state, where the provision of health care services with privacy resulted in a significant positive perception of service quality [31]. This implies that clients will be comfortable when medical staff maintains their privacy. Also, clients believe in emotional attachments, and maintaining privacy makes them satisfied with the service they have obtained [32].

### Limitation of the study

Social desirability bias is likely in our study as the study participants were interviewed in the hospital compound. This might be suffering from response bias that produces more positive responses from the respondents than usual, and this may compromise the result. To reduce this, data collectors and supervisors were recruited from nearby health centers, and participants were thoroughly informed about the study's rationale at each stage of data collection; however, the effect may still occur.

## Conclusion

The overall perceived quality of the present study is poor. The majority of the study participants rated perceived quality as poor. The study showed that waiting time, availability of prescribed drugs at the hospital pharmacy, information on diagnoses (illness), and provision of service with privacy were predictors for client perceived quality. Furthermore, tangibility is the predominant and most important domain of client-perceived quality in public hospitals of Dawro zone. The regional health bureau and zonal health department should understand the issue and work with hospitals to improve outpatient service quality by providing necessary medication, reducing wait times, and designing job training for health care providers**.**

## Data Availability

Data will be available upon request from the corresponding author.

## Abbreviations

EDHS: Ethiopian Demographic Health Survey
FMOH: Federal Ministry of Health
LMICS: Low and Middle Income Countries
OPD: Out Patient Department
SERVQUAL: Service Quality Model
SPPS: Statistical Package for Social Science
WHO: World Health Organization
